# MdERF1B–MdMYC2 module integrates ethylene and jasmonic acid to regulate the biosynthesis of anthocyanin in apple

**DOI:** 10.1093/hr/uhac142

**Published:** 2022-06-23

**Authors:** Shuo Wang, Li-Xian Li, Yue Fang, Dan Li, Zuolin Mao, Zihao Zhu, Xue-Sen Chen, Shou-Qian Feng

**Affiliations:** State Key Laboratory of Crop Biology, College of Horticulture Science and Engineering, Shandong Agricultural University, Tai’an, Shandong 271018, China; State Key Laboratory of Crop Biology, College of Horticulture Science and Engineering, Shandong Agricultural University, Tai’an, Shandong 271018, China; State Key Laboratory of Crop Biology, College of Horticulture Science and Engineering, Shandong Agricultural University, Tai’an, Shandong 271018, China; State Key Laboratory of Crop Biology, College of Horticulture Science and Engineering, Shandong Agricultural University, Tai’an, Shandong 271018, China; State Key Laboratory of Crop Biology, College of Horticulture Science and Engineering, Shandong Agricultural University, Tai’an, Shandong 271018, China; State Key Laboratory of Crop Biology, College of Horticulture Science and Engineering, Shandong Agricultural University, Tai’an, Shandong 271018, China; State Key Laboratory of Crop Biology, College of Horticulture Science and Engineering, Shandong Agricultural University, Tai’an, Shandong 271018, China; State Key Laboratory of Crop Biology, College of Horticulture Science and Engineering, Shandong Agricultural University, Tai’an, Shandong 271018, China

## Abstract

Ethylene and jasmonic acid (JA) are crucial hormones that promote anthocyanin synthesis in apple (*Malus* × *domestica*)*.* However, the mechanism by which these hormones cooperate to modulate anthocyanin production in apple is unclear. According to our results, *MdERF1B* expression was strongly induced by ethylene and JA. Physiological phenotypes and the results of molecular biological analyses indicated that *MdERF1B* encodes a positive regulator of anthocyanin synthesis. Specifically, MdERF1B was capable of combining directly with the *MdMYC2* promoter to promote gene expression. Additionally, MdERF1B interacted with two JA signaling pathway inhibitors, namely MdJAZ5 and MdJAZ10. The MdERF1B–MdJAZ5/10 protein complex decreased the ability of MdERF1B to activate the *MdMYC2* promoter. Furthermore, MdEIL1, which is a crucial protein for ethylene signal transduction, was observed to bind directly to the *MdERF1B* promoter, thereby upregulating gene expression. These results suggest that *MdERF1B* is a core gene responsive to JA and ethylene signals. The encoded protein, together with MdMYC2, MdJAZ5/10, and MdEIL1, modulates anthocyanin synthesis in apple. This study clarifies the synergistic mechanism by which JA and ethylene regulate anthocyanin production in apple.

## Introduction

Anthocyanins, which comprise a subclass of flavonoids widespread among plants, are produced in reactions catalyzed by chalcone isomerase, chalcone synthase, UDP-glucose/flavonoid 3-*O*-glucosyltransferase, dihydroflavonol 4-reductase, and flavanone 3-hydroxylase [1, 2]. Anthocyanin synthesis is regulated by many genes, including those encoding WD40, MYB, and bHLH transcription factors (TFs), which combine to form a regulatory complex that controls anthocyanin production [3–5]. However, anthocyanin synthesis is also regulated by environmental factors (e.g. temperature and light), nutrients (e.g. nitrogen), and hormones [e.g. ethylene (ET) and jasmonic acid (JA)] [ 6–11]. The biological effects of the gaseous hormone ET mainly depend on the transduction of ET signals. ET combines with receptor proteins, such as ET response sensor (ERS) 1, ERS2, ET receptor (ETR) 1, ETR2, and ET insensitive (EIN) 4, to transmit a signal to constitutive triple response 1 (CTR1). The resulting inactivation of CTR1 initiates downstream signals. In addition, EIN2 positively regulates the ET pathway, thereby contributing to endoplasmic reticulum-to-nuclear transmission of the ET signal. Subsequently, the ET signal is transmitted to EIN3 (a primary TF) and triggers the ET response factor (ERF; a secondary TF) [12–16], which ultimately causes activation of downstream ET response genes and regulation of multiple physiological processes, including fruit ripening and anthocyanin accumulation [17, 18].

JA is a relatively recently recognized plant hormone. After it is synthesized, JA binds to isoleucine (Ile) to form JA–Ile [19], which promotes formation of the Skp1–Cul1–coronatine insensitive 1 (F-box protein) complex, which recruits jasmonate ZIM-domain (JAZ) proteins for ubiquitination-based degradation [20, 21]. A consequence of this degradation is the release of several TFs that interact with JAZ proteins, including MYC2, MYB21/MYB24, and EIN3 [22–24]. These TFs further activate downstream genes, while also inducing diverse JA responses, including the regulation of floral development, fruit maturation, and anthocyanin synthesis [23, 25, 26].

ET and JA have synergistic or antagonistic effects on plant growth and developmental regulation, pathogen defense, and fruit ripening [24, 26–28]. For example, JA inhibits the *HLS1* expression activated by ET, thereby inhibiting apical hook formation [27]. The EIN3/EIN3-like 1 (EIL1) proteins integrate ET and JA signals to promote root hair growth and defense responses in plants [24]. Recently, Li and colleagues [26] reported that MdMYC2 mediates JA-induced *MdERF3* expression and ET synthesis to promote apple ripening.

Color is a critical trait of apple fruit quality. Apple fruit coloration mainly depends on anthocyanin accumulation in the peel [29]. Both JA and ET strongly induce anthocyanin synthesis in apple fruit, and thus are critical factors influencing apple fruit color [9]. More specifically, ET activates the expression of anthocyanin production-associated genes via signal transduction factors, such as *MdEIL1*, *MdERF3*, and *MdERF1B*, leading to enhanced anthocyanin synthesis [30–32]. During JA-promoted anthocyanin synthesis, the JA signal transduction factor *MdMYC2* functions as a positive regulator [25, 33]. In contrast, some JAZ proteins, including MdJAZ1 and MdJAZ18, are negative regulators [34, 35]. Previous studies have explored the mechanism by which JA and ET modulate anthocyanin production in apple. However, the relationship of ET with JA during anthocyanin regulation in apple as well as the responsible molecular mechanism remain unclear.

The present research determined that *MdERF1B* is a core gene responsive to JA and ET signals. The encoded protein, as well as MdMYC2, MdJAZ5/10, and MdEIL1, modulate anthocyanin synthesis in apple. On the basis of these findings, we elucidated a new regulatory mechanism by which MdERF1B promotes anthocyanin synthesis, and provide researchers with useful insights into the interrelation between JA and ET during the regulation of apple anthocyanin synthesis.

## Results

### Both ethylene and jasmonic acid promote expression of *MdERF1B* and *MdMYC2* as well as anthocyanin synthesis in apple fruit

**Figure 1:**
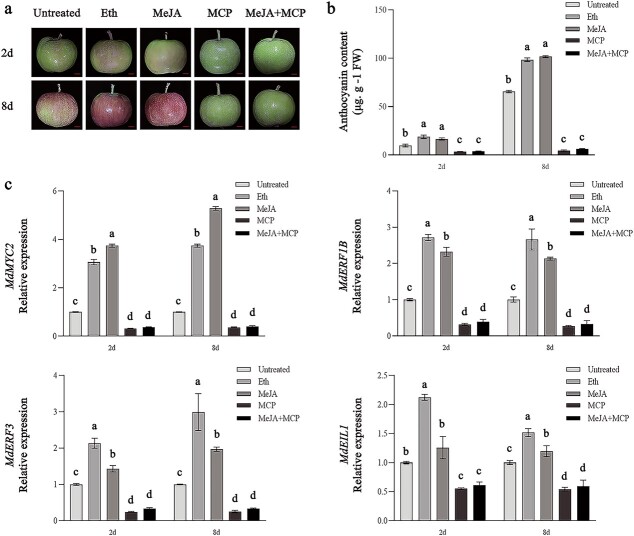
Ethephon and MeJA induce anthocyanin accumulation and *MdMYC2*, *MdERF1B*, and *MdERF3* expression in fruits of ‘Geneva Early’ apple. **a** Fruits exposed to ethephon, MeJA, 1-MCP, or MeJA + 1-MCP under light (20 000 lux) at 24°C for 2 and 8 days. Scale bars represent 1 cm. **b** Anthocyanin content in fruits treated with 1-MCP, MeJA, ethephon, or MeJA +1-MCP and control fruits. **c***MdMYC2*, *MdERF1B*, *MdERF1*, and *MdEIL1* expression levels in fruits treated with 1-MCP, MeJA, ethephon, or MeJA +1-MCP and control fruits. Control, untreated fruits; Eth, fruits exposed to ethephon; MeJA, fruits exposed to MeJA; MCP, fruits treated with 1-MCP. Error bars represent the standard error from three individual assays. Different letters represent significant differences (*P* < .05, Tukey’s test).

Both ET and JA can promote anthocyanin synthesis and apple fruit coloration, but the combined regulatory effects of ET and JA on anthocyanin synthesis and the molecular mechanism responsible are uncharacterized. To further explore the regulation of anthocyanin synthesis in apples by ET and JA, fruits of ‘Geneva Early’ apples were collected 60 days after full bloom (DAFB) for the following treatments: ethephon solution (1000 mg L^−1^), 1-methylcyclopropene (1-MCP, 1 μL L^−1^, ET inhibitor), methyl jasmonate (MeJA; 100 μM), and MeJA (100 μM) + 1-MCP (1 μL L^−1^). After treatment, fruits were preserved under 24°C and constant light (20 000 lux). We selected fruits stored after 2 and 8 days for analysis of gene expression and anthocyanin contents. Compared with the control, the ethephon and MeJA treatments significantly increased anthocyanin content in the fruit peel. Interestingly, treatment with MeJA + 1-MCP reduced anthocyanin accumulation in the fruit peel (Fig. 1a and b), implying that JA-induced anthocyanin production is dependent on ET production.

The JA signaling pathway gene *MdMYC2* and the ET signaling pathway genes *MdEIL1*, *MdERF1B*, and *MdERF3* can regulate anthocyanin synthesis in apple [25, 30–32]. To further functionally characterize these genes in terms of their regulatory effects on ET- and JA-induced anthocyanin synthesis, we analyzed their expression in the treated fruits. The *MdMYC2*, *MdEIL1*, *MdERF1B*, and *MdERF3* expression levels were significantly higher in the fruits exposed to ethephon than in the control fruits. In addition, the expression of *MdMYC2*, *MdERF1B*, and *MdERF3* significantly increased among MeJA-treated fruits compared with control fruits. In contrast, *MdMYC2*, *MdERF1B*, and *MdERF3* were expressed at lower levels in fruits exposed to 1-MCP or MeJA + 1-MCP than in the control fruits (Fig. 1c). These observations suggested that ET- and JA-induced *MdMYC2*, *MdERF1B*, and *MdERF3* expression may be critical for anthocyanin synthesis in apple. Previous studies have confirmed that *MdERF3* is a target gene directly regulated by MdMYC2 [26]. Therefore, we selected *MdMYC2* and *MdERF1B* for an in-depth study of their functions during ET- and JA-regulated anthocyanin synthesis.

### *MdERF1B* and *MdMYC2* positively regulate anthocyanin production in apple fruits

**Figure 2:**
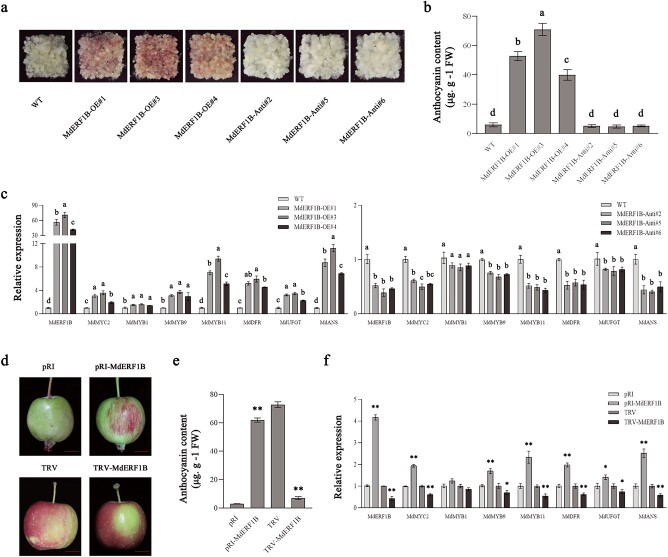
Analysis of the physiological function of *MdERF1B*. **a**–**c***MdERF1B* overexpression increased anthocyanin biosynthesis in apple calli. Phenotypes (**a**), anthocyanin contents (**b**), and expression levels of *MdERF1B*, *MdMYC2*, and genes related to anthocyanin biosynthesis (**c**) in *MdERF1B*-OE (*MdERF1B*-overexpressing) and *MdERF1B*-Anti (*MdERF1B*-antisense) lines. WT, wild-type control. **d–f** Apple ‘Otome’ fruits were sampled at 50 and 140 DAFB and subsequently injected with the antisense viral vector TRV-*MdERF1B* or the overexpression vector pRI-*MdERF1B*. Phenotypes (**d**), anthocyanin contents (**e**), and expression levels of *MdERF1B*, *MdMYC2*, and genes associated with anthocyanin production (**f**) in the pericarp around the injection site. FW, fresh weight. Scale bar = 1 cm. Error bars represent the standard error of three independent assays. Different letters represent significant differences (*P* < .05, Tukey’s test). ^*^P < .05, ^**^P < .01 (Student’s *t*-test).

We generated transgenic apple calli in which *MdERF1B* and *MdMYC2* were overexpressed or silenced. These calli were cultured at 16°C under constant light (20 000 lux). Compared with control calli, *MdERF1B* overexpression significantly enhanced anthocyanin production as well as the expression of *MdMYB9*, *MdDFR*, *MdMYB11*, *MdANS*, and *MdUFGT* in the transgenic calli; however, the silencing of *MdERF1B* reduced anthocyanin production and the expression of *MdDFR*, *MdMYB9*, *MdANS*, and *MdMYB11* in the transgenic calli (Figure 2a–c). In comparison with the control calli, *MdMYC2* expression was markedly higher in the *MdERF1B*-overexpressing (OE) transgenic calli, whereas it was significantly lower in transgenic calli harboring the RNA interference (RNAi) construct *MdERF1B*-RNAi (Figure 2c). In addition, we injected apple fruits with the TRV-*MdERF1B* silencing vector or the pRI-*MdERF1B* overexpression vector. Compared with the control fruits, transient *MdERF1B* overexpression increased anthocyanin production and *MdMYB9*, *MdMYB11*, *MdDFR*, *MdUFGT*, and *MdANS* expression levels in the peel at the injection site. The transient silencing of *MdERF1B* inhibited anthocyanin production and the associated gene expression (Figure 2d–f). Subsequently, we analyzed the expression of *MdMYC2* at the injection site in the peel. Compared with the control fruits, the *MdMYC2* expression level was higher in the peel of *MdERF1B*-OE fruits, whereas it was lower in the *MdERF1B*-RNAi fruits (Figure 2f).

**Figure 3:**
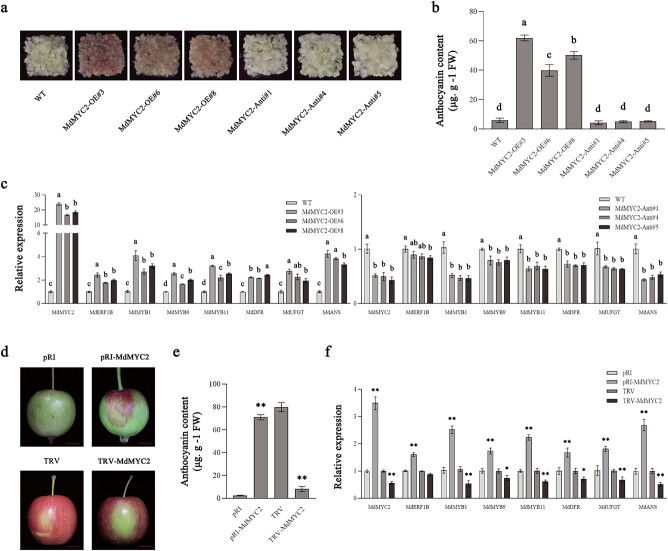
Analysis of the physiological function of *MdMYC2*. **a–c***MdMYC2* overexpression enhanced anthocyanin biosynthesis in apple calli. Phenotypes (**a**), anthocyanin contents (**b**), and expression levels of *MdMYC2*, *MdERF1B*, and anthocyanin biosynthetic genes (**c**) in *MdMYC2*-OE (*MdMYC2*-overexpressing) and *MdMYC2*-Anti (*MdMYC2*-antisense) lines. WT, wild-type control. **d–f** Apple ‘Otome’ fruits were sampled at 50 and 140 DAFB and then injected with the antisense viral vector TRV-*MdMYC2* or the overexpression vector pRI-*MdMYC2*. Phenotypes (**d**), anthocyanin contents (**e**), and expression levels of *MdMYC2*, *MdERF1B*, and genes associated with anthocyanin production (**f**) in the pericarp around the injection site. FW, fresh weight. Scale bar = 1 cm. Error bars represent the standard error from three independent assays. Different letters represent significant differences (*P* < .05, Tukey’s test). ^*^P < .05, ^**^P < .01 (Student’s *t*-test).

Compared with the levels in the corresponding control, the overexpression of *MdMYC2* increased anthocyanin production and upregulated *MdMYB1*, *MdMYB9*, *MdMYB11*, *MdDFR*, *MdUFGT*, and *MdANS* expression in the transgenic calli. By contrast, the silencing of *MdMYC2* reduced anthocyanin production and the related gene expression in the transgenic calli (Figure 3a–c). In addition, we subsequently analyzed *MdERF1B* expression in the transgenic calli in which *MdMYC2* was overexpressed or silenced. Relative to controls, *MdERF1B* expression level markedly increased in *MdMYC2*-OE transgenic calli, but the difference was not significant in *MdMYC2*-RNAi transgenic calli compared with controls (Figure 3c). We injected apple ‘Otome’ fruits with the TRV-*MdMYC2* silencing vector and the pRI-*MdMYC2* overexpression vector. In comparison with the control levels, the transient overexpression of *MdMYC2* enhanced anthocyanin production and *MdMYB1*, *MdMYB9*, *MdMYB11*, *MdDFR*, *MdUFGT*, and *MdANS* expression at the injection site of the peel. By contrast, the transient silencing of *MdMYC2* inhibited anthocyanin biosynthesis as well as expression levels of relevant genes (Figure 3d–f). Additionally, *MdERF1B* expression at the injection site of the peel was examined, which revealed that this gene was more highly expressed in the *MdMYC2-*OE peel than in the control peel (Figure 3f). No significant difference in *MdERF1B* expression between the *MdMYC2*-RNAi and control peels was observed. These results were consistent with those obtained for the transgenic calli. These findings are suggestive of a direct regulatory relationship between *MdERF1B* and *MdMYC2*.

**Figure 4:**
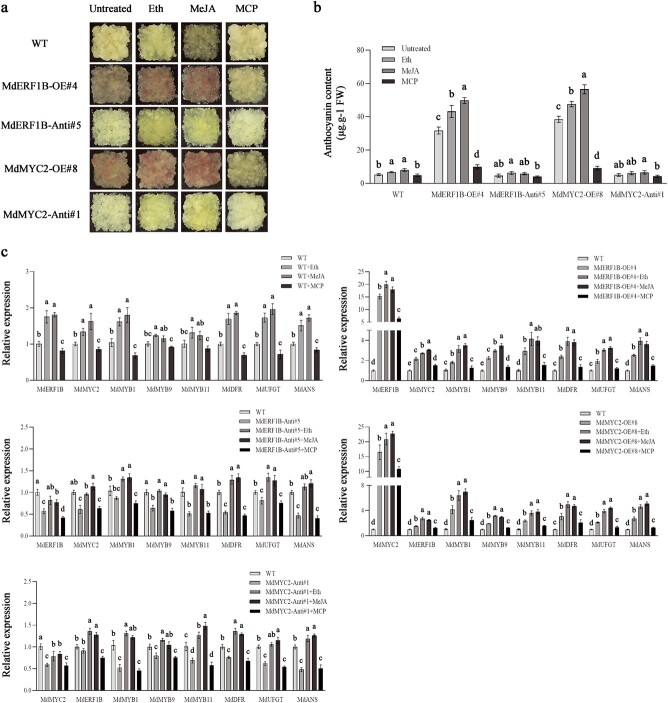
Ethephon and MeJA promote anthocyanin accumulation triggered by *MdERF1B* and *MdMYC2*. **a–c** Phenotypes (**a**), anthocyanin contents (**b**), and expression levels of *MdMYC2*, *MdERF1B*, and genes associated with anthocyanin production (**c**) in apple calli exposed to ethephon, MeJA, or 1-MCP. WT, wild-type control; *MdMYC2*-OE, *MdMYC2*-overexpressing calli; *MdMYC2*-Anti, *MdMYC2*-antisense calli; *MdERF1B*-Anti, *MdERF1B*-antisense calli; *MdERF1B*-OE, *MdERF1B*-overexpressing calli. Control, untreated fruits; Eth, ethephon-exposed fruits; MeJA, MeJA-exposed fruits; MCP, 1-MCP-exposed fruits. Error bars represent the standard error from three independent assays. Different letters represent significant differences (*P* < .05, Tukey’s test).

Additionally, the transgenic apple calli *MdERF1B*-OE#4, *MdERF1B*-Anti#5, *MdMYC2*-OE#8, and *MdMYC2*-Anti#1 were exposed to 1-MCP (1 μL L^−1^), MeJA (100 μM), or ethephon (1000 mg L^−1^). Overexpression of *MdERF1B* and *MdMYC2* promoted MeJA- and ethephon-induced anthocyanin production. In contrast, the silencing of *MdERF1B* and *MdMYC2* prevented this promotion of anthocyanin production (Fig. 4a and b). In comparison with the control calli, *MdMYC2* was more highly expressed in the *MdERF1B-*OE apple calli treated with MeJA or ethephon, whereas *MdMYC2* expression was reduced in the *MdERF1B*-RNAi transgenic calli treated with MeJA or ethephon, indicating that *MdMYC2* plays a role in *MdERF1B*-mediated anthocyanin accumulation (Fig. 4c).

### MdERF1B binds to *MdMYC2* promoter to trigger expression

**Figure 5:**
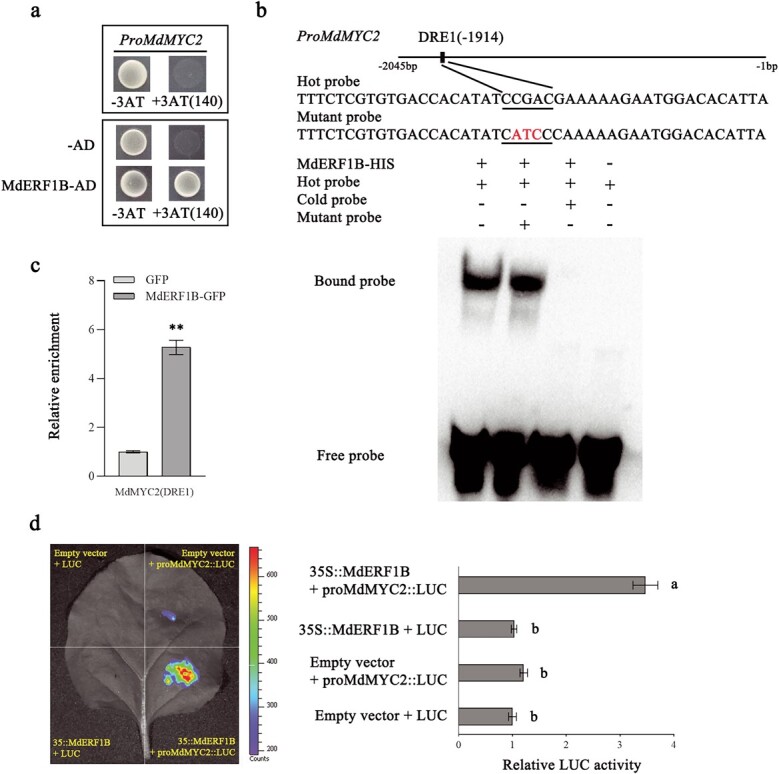
MdERF1B can bind to the *MdMYC2* promoter and increase transcription. **a** Based on Y1H experimental findings, MdERF1B bound to the *MdMYC2* promoter. We used 140 mM 3-AT. Both the *MdMYC2* promoter and empty pGAD vector served as negative controls. **b** EMSA results suggested that MdERF1B can bind to the *MdMYC2* promoter’s DRE1 motif. The DRE1 motif-containing biotin-conjugated *MdMYC2* promoter fragment was used as a hot probe, while the unmarked competitive probe (300-fold relative to hot probe) was used as a cold probe. This work used the unmarked hot probe that included three mutated nucleotides as the mutant probe. **c** Based on ChIP–qPCR assay results, MdERF1B could bind to the *MdMYC2* promoter *in vivo*. Apple calli overexpressing GFP were employed as a negative control. Three biological replicates were performed for the ChIP assay. **d** According to the LUC reporter gene assay, MdERF1B promoted *MdMYC2* promoter activity in tobacco leaves. Error bars represent the standard error from three individual assays. ^**^P < .01 (Student’s *t*-test). Different letters represent significant differences (*P* < .05, Tukey’s test).

We clarified the regulatory relationship between *MdERF1B* and *MdMYC2* by performing the yeast one-hybrid (Y1H) experiment. Yeast strain Y187 cells harboring pGAD-*MdERF1B* and pHIS2-pro*MdMYC2* grew on the corresponding prepared (SD/−Trp/−Leu/−His) media that contained 3-amino-1,2,4-triazole (3-AT), whereas yeast Y187 cells co-transformed with pGAD-*MdMYC2* and pHIS2-pro*MdERF1B* did not grow on these media (Figure 5a, Supplementary Data Fig. S1). Accordingly, MdERF1B was able to interact with the *MdMYC2* promoter, whereas MdMYC2 was unable to interact with the *MdERF1B* promoter. We detected three potential MdERF1B-binding elements (CCGAC; DRE1–3) in the *MdMYC2* promoter. We conducted the electrophoretic mobility shift assay (EMSA) to test the ability of MdERF1B to interact with the DRE1–3 motifs in the *MdMYC2* promoter. When an unlabeled DNA fragment (i.e. cold probe) containing DRE1 was added as a competitor, the band consistent with the analyzed interaction was not detected. In contrast, the band was unaffected by the addition of an unlabeled DNA fragment containing a mutated DRE1 element as a competitor (Figure 5b). Hence, MdERF1B bound specifically to the DRE1 motif in the *MdMYC2* promoter. However, the EMSA results revealed that MdERF1B was unable to bind to DRE2 or DRE3 in the *MdMYC2* promoter *in vitro* (Supplementary Data Fig. S2).

To examine whether MdERF1B is able to bind to the *MdMYC2* promoter *in vivo*, we used transgenic apple calli overexpressing green fluorescent protein (GFP)-labeled MdERF1B to perform a chromatin immunoprecipitation and quantitative PCR (ChIP–qPCR) assay. Apple calli overexpressing GFP alone served as the control. The *MdMYC2* promoter fragments containing DRE1 were significantly more enriched in *MdERF1B*-OE calli compared with controls (Figure 5c), implying that MdERF1B is able to bind to DRE1 in the *MdMYC2* promoter *in vivo*.

To assess how MdERF1B affects *MdMYC2* promoter activity, we used *Agrobacterium tumefaciens* to transiently transform *Nicotiana benthamiana* leaves for luciferase (LUC) reporter gene assays. We fused the *MdMYC2* promoter sequence to the LUC reporter gene of pGreenII 0800-LUC vector. We also inserted the *MdERF1B* coding sequence (CDS) into pGreenII 62-SK vector for use as the effector. Tobacco leaves were co-injected with the recombinant plasmids and then examined. Fluorescence intensity was significantly higher for leaves transfected with 35S::*MdERF1B* and pro*MdMYC2*:LUC than for leaves transfected with pro*MdMYC2*:LUC alone or control leaves (Figure 5d). Thus, MdERF1B was indicated to increase the *MdMYC2* promoter activity.

### MdMYC2 is crucial for *MdERF1B*-regulated anthocyanin synthesis

**Figure 6:**
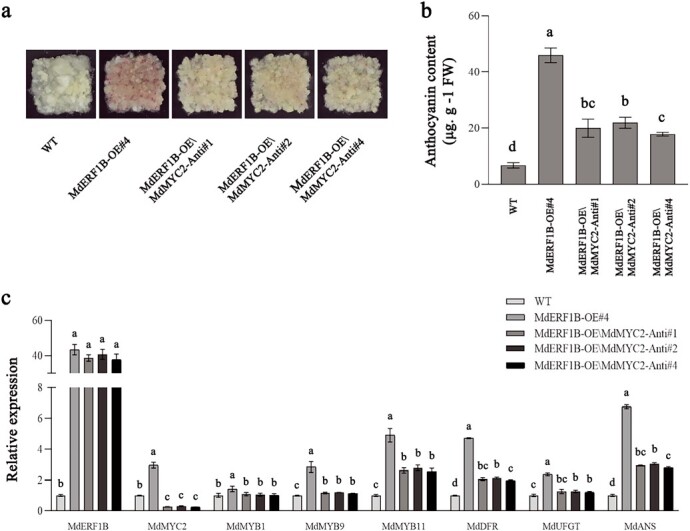
*MdERF1B* enhances anthocyanin production through *MdMYC2*. **a** Apple callus phenotype. **b** Anthocyanin contents in apple calli. FW, fresh weight. **c** Anthocyanin production-related gene expression in apple calli. WT, wild-type control; *MdERF1B*-OE, *MdERF1B*-overexpressing calli; *MdMYC2*-Anti, *MdMYC2*-antisense calli. Error bars represent the standard error of three individual assays. Different letters represent significant differences (*P* < .05, Tukey’s test).

To explore *MdMYC2*’s function in *MdERF1B*-regulated anthocyanin production, we constructed the pFGC1008-*MdMYC2* recombinant plasmid and inserted it into *MdERF1B*-OE apple calli to produce genetically modified apple calli in which *MdMYC2* expression was reduced [i.e. *MdMYC2*-antisense (Anti) calli]. The anthocyanin content in the *MdMYC2*-Anti calli was higher compared with that in the control calli, but was markedly lower than that in the *MdERF1B*-OE calli (Fig. 6a and b). In addition, anthocyanin-associated gene expression in the transgenic calli was explored. The expression of *MdMYB9*, *MdMYB11*, *MdDFR*, *MdANS*, and *MdUFGT* increased in *MdMYC2*-Anti calli compared with wild-type controls, but was highest in the *MdERF1B*-OE calli (Fig. 6c). Collectively, these findings indicated that *MdMYC2* is important for *MdERF1B*-regulated anthocyanin synthesis.

### MdEIL1 can bind to *MdERF1B* promoter and induces expression

**Figure 7:**
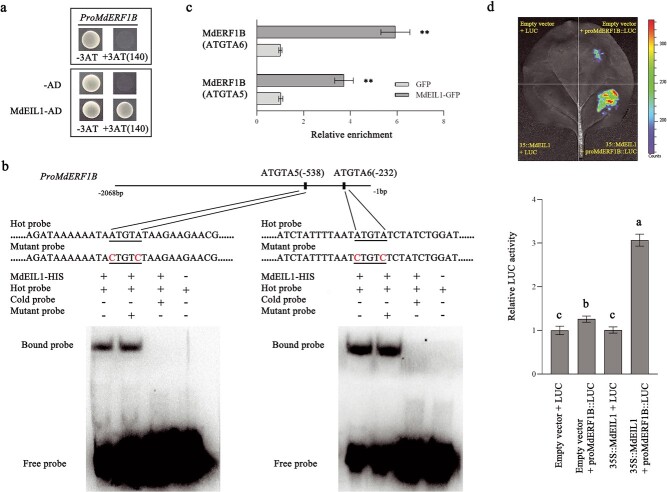
MdEIL1 binds to *MdERF1B* promoter. **a** Y1H experimental results indicate the binding of MdEIL1 to *MdERF1B* promoter. We used 140 mM 3-AT. The *MdERF1B* promoter and empty pGAD vector were used as negative controls. **b** According to EMSA results, MdEIL1 bound to ATGTA5 and ATGTA6 within *MdERF1B* promoter. This work used the biotin-conjugated *MdERF1B* promoter fragment including ATGTA5 and ATGTA6 as the hot probe, while the cold probes were unlabeled identical DNA fragments used as competitors (300-fold relative to the hot probe). The unmarked hot probe containing two mutated nucleotides in ATGTA served as the mutated probe. **c** ChIP–qPCR assay results showing that MdEIL1 is capable of binding to the *MdERF1B* promoter *in vivo*. Apple calli overexpressing GFP were employed as the negative control. Three biological replicates were performed for the ChIP analysis. **d** LUC reporter gene assays showing that MdEIL1 increases *MdERF1B* promoter activities in tobacco leaves. Error bars represent the standard error from three independent assays. ^**^P < .01 (Student’s *t*-test). Different letters represent significant differences (*P* < .05, Tukey’s test).

To further investigate the *MdERF1B* response to ET, we analyzed the regulatory relationship between *MdEIL* [30] and *MdERF1B*. The *MdERF1B* expression level increased in *MdEIL1*-OE calli compared with wild-type calli (Supplementary Data Fig. S3). Interaction of MdEIL1 with the *MdERF1B* promoter was subsequently confirmed by a Y1H assay (Fig. 7a). Six possible MdEIL1-binding elements (ATGTA1–6) were detected in the *MdERF1B* promoter. Hence, we analyzed the association of MdEIL1 with the *MdERF1B* promoter by conducting the EMSA. The results showed that MdEIL1 can bind to ATGTA5 and ATGTA6 within the *MdERF1B* promoter (Fig. 7b, Supplementary Data Fig. S4). Furthermore, we performed a ChIP–qPCR analysis to verify the *in vivo* binding of MdEIL1 to the *MdERF1B* promoter. The overexpression of *MdEIL1* markedly increased the abundance of *MdERF1B* promoter fragments containing ATGTA5 and ATGTA6 in the immunoprecipitation samples (Fig. 7c). Therefore, MdEIL1 was capable of binding to the *MdERF1B* promoter *in vivo*. Transient LUC reporter gene assays showed that MdEIL1 significantly increased the *MdERF1B* promoter activity (Fig. 7d). Thus, MdEIL1 can upregulate the *MdERF1B* level by combining with the *MdERF1B* promoter.

### MdERF1B interacts with MdJAZ5 and MdJAZ10

**Figure 8:**
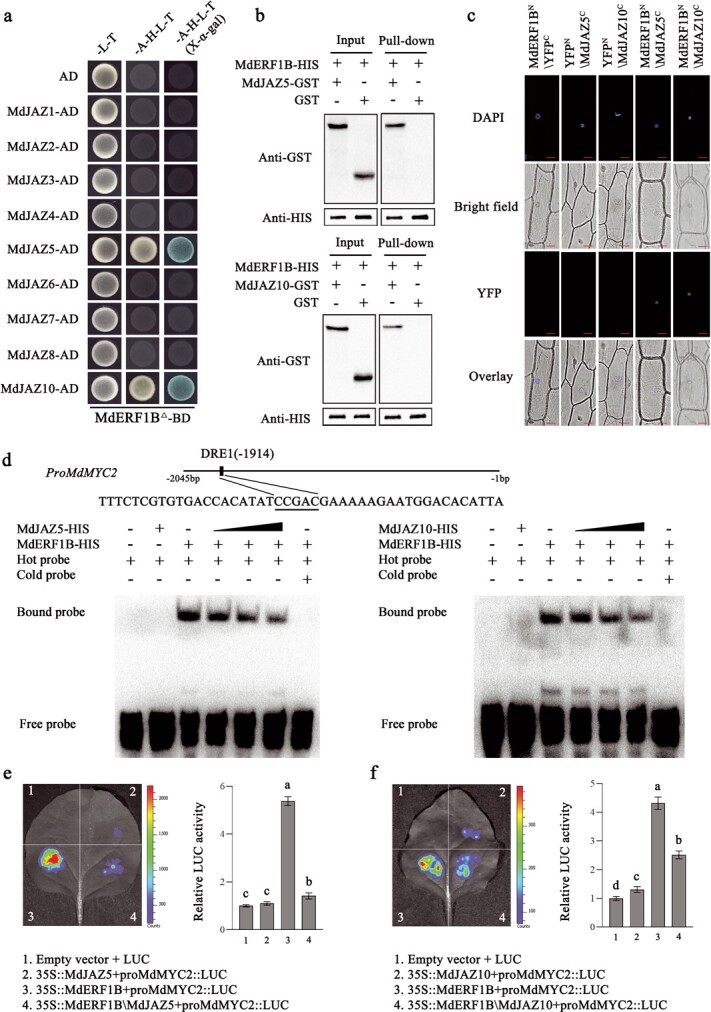
MdERF1B shows interaction with MdJAZ5 and MdJAZ10. **a** Y2H experiments confirming that MdERF1B interacted with MdJAZ5 and MdJAZ10. This work employed pGAD vector as the negative control. Blue plaques signify a protein interaction. **b** Pull-down assays suggesting that MdERF1B interacted with MdJAZ5 and MdJAZ10 *in vitro*. We used anti-histidine and anti-glutathione *S*-transferase antibodies for western blot analyses. **c** Bimolecular fluorescence complementation assay results demonstrating that MdERF1B interacts with MdJAZ5 and MdJAZ10. This work used YFPN together with YFPC empty vectors as negative controls. Scale bars represent 50 μm. **d** MdJAZ5/10–MdERF1B interaction inhibited the binding of MdERF1B to the *MdMYC2* promoter. EMSA results revealed that MdJAZ5 and MdJAZ10 do not bind to the DRE1 site in the *MdMYC2* promoter, but MdERF1B binds to this motif. Both MdJAZ5 and MdJAZ10 interfered with the binding of MdERF1B to the *MdMYC2* promoter. This work used the DRE1 motif-containing biotin-conjugated *MdMYC2* promoter fragment as hot probe, while unmarked competitive probe (300-fold in relation to hot probe) was the cold probe. The mutant probe was an unmarked hot probe that included three mutated nucleotides. **e**, **f** LUC reporter gene assays revealing that the interaction between MdERF1B and MdJAZ5 (**e**) as well as MdJAZ10 (**f**) attenuated the transcriptional activation impact of MdERF1B on the *MdMYC2* promoter. Error bars represent the standard error of three separate assays. Different letters represent significant differences (*P* < .05, Tukey’s test).

To clarify the mechanism for the response of MdERF1B to JA, we used MdERF1B^Δ^ (i.e. MdERF1B without the autoactivation domain) as bait for the yeast two-hybrid (Y2H) experiment conducted to select interactive proteins from among MdJAZ proteins (MdJAZ1–8 and MdJAZ10) [34] and MdMYC2 [26]. Yeast cells harboring pGBK-*MdERF1B^Δ^* and pGAD-*MdJAZ5*/*10* were able to grow on SD/−Trp/−Leu/−His/−Ade media supplemented with X-α-galactose (Fig. 8a). By contrast, yeast cells harboring pGAD-*MdMYC2* and other pGAD-*MdJAZ* recombinant plasmids did not grow (Supplementary Data Fig. S5). These observations indicated that MdERF1B was able to interact with MdJAZ5 and MdJAZ10, but not with MdMYC2 or the other MdJAZ proteins. In the subsequent pull-down assay, MdJAZ5/10-GST was pulled down by MdERF1B-HIS, suggesting that MdERF1B and MdJAZ5/10 interacted *in vitro* (Fig. 8b). In addition, signals of yellow fluorescent protein (YFP) could be observed in the onion epidermal cells with co-expression of MdERF1B-YFPN with MdJAZ5/10-YFPC (Fig. 8c). Bimolecular fluorescence complementation assay results confirmed that MdERF1B was able to interact with MdJAZ5/10 *in vivo*. As mentioned above, MdERF1B is a transcriptional activator that can bind to the *MdMYC2* promoter with the purpose of inducing expression. Therefore, an EMSA was performed to test whether MdERF1B–MdJAZ5 or MdERF1B–MdJAZ10 interaction affected MdERF1B’s binding to the *MdMYC2* promoter. Both MdJAZ5 and MdJAZ10 were unable to bind to the DRE1 site within the *MdMYC2* promoter. Binding of MdERF1B to the *MdMYC2* promoter progressively weakened when MdJAZ5 and MdJAZ10 increased (Fig. 8d). This observation reflected the inhibitory effects of MdJAZ5/10–MdERF1B on the binding of MdERF1B to the *MdMYC2* promoter. To assess whether the MdERF1B–MdJAZ5/10 protein complex influences the regulatory effects of MdERF1B on *MdMYC2*, we performed a transient LUC reporter gene assay. The *MdMYC2* promoter activity in response to the co-expression of MdJAZ5/10 and MdERF1B was significantly lower than that following the expression of MdERF1B alone (Fig. 8e and f), indicating that the interaction between MdJAZ5/10 and MdERF1B significantly decreased the ability of MdERF1B to activate the *MdMYC2* promoter.

## Discussion

Both ET and JA are important hormones that regulate apple fruit ripening, especially coloration [9]. In addition, MdEIL1, MdERF3, and MdERF1B, which are ET signal transducers, as well as MdMYC2, which is a JA signal transducer, actively regulate anthocyanin synthesis in apple [25, 30–32]. In contrast, MdJAZ1 and MdJAZ18, which are JA signaling inhibitors, adversely affect anthocyanin synthesis in apple [34, 35]. According to the present results, *MdMYC2*, *MdERF1B*, and *MdERF3* expression levels were substantially upregulated by JA and ET, suggesting that the encoded proteins function as important regulators of JA- and ET-induced anthocyanin production in apple. Therefore, the molecular mechanism responsible for the effects of these proteins on JA- and ET-induced anthocyanin production in apple should be characterized.

The *MdMYC2* gene encodes a critical regulator of the JA pathway, whose level is upregulated through JA [36]. Recently, according to Li and colleagues [26], expression of *MdMYC2* can be induced by JA in apple and activates the expression of the MdERF3 target gene *MdACS1* by upregulating the transcription of *MdERF3*, thereby promoting ET synthesis and fruit ripening. In the present study, *MdMYC2*/*MdERF3* had the same expression trend upon MeJA exposure (i.e. expression of both genes was upregulated). This finding supports the view that JA upregulates *MdERF3* expression via MdMYC2. Furthermore, the ethephon treatment upregulated *MdMYC2* expression, implying that ET may also upregulate *MdERF3* expression through MdMYC2. Given that MdERF3 can induce anthocyanin-related gene expression and anthocyanin synthesis, we speculated that JA and ET can promote anthocyanin biosynthesis synergistically via the *MdMYC2*–*MdERF3* pathway.

The mechanism by which ET induces *MdMYC2* expression is unclear. On the basis of the present findings, MdERF1B, which is an ET signal transducer, can bind directly to the *MdMYC2* promoter to increase expression. Therefore, the ET signal indirectly upregulates the expression of *MdMYC2* via MdERF1B. The ET–*MdERF1B*–*MdMYC2* pathway revealed in the present study and the JA–*MdMYC2*–*MdERF3*–*MdACS1*–ET pathway discovered by Li *et al*. [26] form a JA and ET regulatory network.

An *et al*. [30] recently reported that the *MdEIL1*–*MdMYB1* module is important for ET-induced anthocyanin synthesis in apple. As reported by Zhang *et al*. [32], MdERF1B promotes *MdMYB1* expression and interacts with the encoded protein to positively regulate anthocyanin synthesis in apple. In the current study, we demonstrated that MdEIL1, the critical ET pathway component, can bind to the *MdERF1B* promoter, thereby increasing expression. Thus, ET can promote anthocyanin synthesis via the *MdEIL1*–*MdERF1B*–*MdMYB1* module.

**Figure 9:**
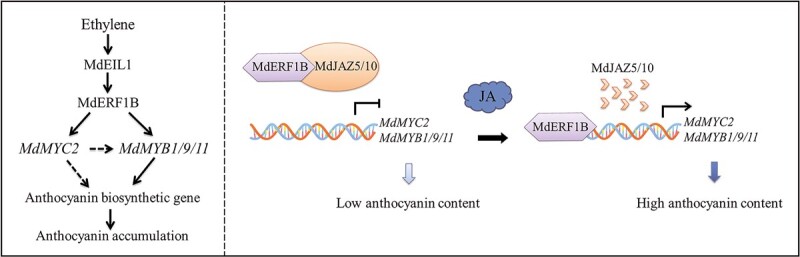
Model for MdERF1B mediating ET- and JA-regulated anthocyanin synthesis in apple. (Left) MdERF1B mediates ET-regulated anthocyanin synthesis. ET-induced MdEIL1 binds to the *MdERF1B* promoter to increase transcription. MdERF1B then directly upregulates *MdMYC2* and *MdMYB1*/*9*/*11* expression, which activates anthocyanin structural genes and anthocyanin accumulation. (Right) MdERF1B mediates JA-regulated anthocyanin synthesis. The physical interaction between MdJAZ5/10 and MdERF1B inhibits *MdMYC2* and *MdMYB1*/*9*/*11* promoter activation, leading to relatively low *MdMYC2* and *MdMYB*1/*9*/*11* expression levels and downregulated levels of genes related to anthocyanin production, thereby inhibiting anthocyanin accumulation. When JA levels increase, MdJAZ5 and 10 are degraded. The resulting free MdERF1B increases *MdMYC2* and *MdMYB1*/*9*/*11* promoter activities, leading to relatively high *MdMYC2* and *MdMYB1*/*9*/*11* expression levels as well as upregulation of genes associated with anthocyanin biosynthesis and accumulation. Solid and dashed arrows represent direct and unclear regulation, respectively.

Previous research indicated that JAZ proteins may inhibit JA responses by interacting with various TFs. Abnormal JAZ protein levels may disrupt such interactions, resulting in the release of TFs that are free to activate various JA-regulated biological processes [20, 37]. For example, JAZ proteins can attenuate the transcriptional regulation mediated by bHLH TFs, such as MYC2/3/4, TT8, GL3, EGL3, and bHLH3, as well as by MYB TFs, including MYB21/24/75, GL1, and telomere repeat-binding protein 1, to suppress JA-modulated root development, stamen growth, and anthocyanin synthesis [22, 23, 25, 34, 35, 38–41]. In the current work, we identified MdERF1B as a novel interacting partner of JAZ proteins. Moreover, MdJAZ5 and MdJAZ10 are able to interact with MdERF1B to restrict its ability to activate *MdMYC2* expression. Both MdJAZ5 and MdJAZ10 were degraded in the presence of MeJA (Supplementary Data Fig. S6), which may result in the release of MdERF1B, and the subsequent induction of *MdMYC2* expression and anthocyanin accumulation. Moreover, *MdJAZ5* and *MdJAZ10* expression was triggered by exogenous MeJA (Supplementary Data Fig. S7), suggesting that expression of both genes might be important in preventing cellular damage caused by JA hyperactive responses. Hence, JA-regulated anthocyanin synthesis is mediated by the interaction between JAZ proteins and MdERF1B. In addition, *MdJAZ5* and *MdJAZ10* expression was triggered by ET, suggesting that the encoded proteins contribute to the ET signaling pathway (Supplementary Data Fig. S7). Further research is needed to elucidate the mechanism of this phenomenon.

We developed a model highlighting the importance of MdERF1B for JA- and ET-induced anthocyanin synthesis in apple. First, MdEIL1 upregulates *MdERF1B* expression in response to ET signals. Next, MdERF1B binds to the *MdMYC2* and *MdMYB1*/*9*/*11* promoters to upregulate expression, ultimately leading to increased anthocyanin synthesis and accumulation. When JA is absent or its concentration is low, MdJAZ5 and MdJAZ10 interact with MdERF1B to weaken its transcriptional activation function, thereby decreasing *MdMYC2* and *MdMYB1*/*9*/*11* expression levels. When the JA concentration increases, JAZ proteins are degraded via the ubiquitination pathway [20], which results in the release of MdERF1B, which then activates *MdMYC2* and *MdMYB1*/*9*/*11* expression. The encoded TFs subsequently induce the expression of the downstream genes associated with anthocyanin synthesis, leading to anthocyanin production (Figure 9).

## Materials and methods

### Plant materials and processing

We collected apple (*Malus* × *domestica*) ‘Geneva Early’ fruits from mature trees growing in Linyi, Shandong, China. We sampled fruits of ‘Geneva Early’ at 60 DAFB then promptly transported them to the laboratory. We classified the fruits into five groups (*n* = 30 in each group). The samples in group 1 were untreated and used as controls. The samples in group 2 were soaked for 1 minute in ethephon solution (1000 mg L^−1^). The surface of the samples in group 3 was exposed to MeJA (100 μM). Group 4 was treated with 1-MCP (1 μL L^−1^) for 12 hours. The samples in group 5 were subjected to 12 hours of treatment with 1-MCP (1 μL L^−1^) and then to treatment with MeJA (100 μM). Subsequently, the samples were placed in a growth chamber (24°C) under constant light (20 000 lux). At 2 and 8 days after treatment, fruits were subjected to analysis of gene expression and anthocyanin contents. The experiments were performed in triplicate, with five fruits per biological replicate.

We collected apple ‘Otome’ fruits from mature trees growing on the laboratory farm of the Shandong Pomology Institute (Taian, Shandong, China). The fruits were sampled at 50 and 140 DAFB for assays of gene overexpression and gene silencing injection, respectively. ‘Orin’ apple calli were cultured in Murashige and Skoog (MS) medium at 25°C in the dark.

### Anthocyanin content determination

In this study, HCl-methanol (1% v/v) was used in the extraction of anthocyanins. In brief, we added 0.5 g plant material ground in liquid nitrogen to 10 mL of 1% HCl-methanol (v/v). The resulting solution was thoroughly mixed and incubated for 1 day at 4°C in the dark. Then, we added 1-mL of extract to each of 4 mL of NaAC (pH 4.5) and 4 mL KCl (pH 1.0), respectively. The solution absorbance (OD) values were determined at 510 and 700 nm using the UV-2450 spectrophotometer (Shimadzu, Kyoto, Japan). The anthocyanin level was determined by the pH differential approach [42].

### RNA extraction and quantification by quantitative reverse transcription–PCR

We used the RNAprep Pure Plant Kit (TianGen, Beijing, China) to extract total RNA. In addition, the RevertAid™ First Strand cDNA Synthesis Kit (TransGen) was employed for cDNA synthesis. We conducted quantitative reverse transcription–PCR (qRT–PCR) analysis on the CFX96 system (Bio-Rad, Hercules, CA, USA) with 20-μL reaction solutions including 1 μL of the respective primers, 1 μL cDNA, and 10 μL SYBR Green Master Mix (TransGen). Supplementary Data Table S1 lists all qRT–PCR primers. The 2^−ΔΔ*C*t^ method [43] was used to determine gene expression based on qRT–PCR data.

### Overexpression of *MdERF1B*, *MdMYC2*, and *MdEIL1* and silencing of *MdERF1B* and *MdMYC2* in ‘Orin’ calli

To overexpress *MdERF1B*, *MdMYC2*, and *MdEIL1* in apple calli, we inserted the full-length *MdERF1B*, *MdMYC2*, and *MdEIL1* CDSs in plasmid pRI101-AN, which contained the GFP tag, to generate overexpression vectors, namely pRI-*MdERF1B*, pRI-*MdMYC2*, and pRI-*MdEIL1*. To silence *MdERF1B* and *MdMYC2* expression in apple calli, we recombined the 342- and 369-bp antisense and sense sequences of *MdERF1B* and *MdMYC2* in the pFGC1008 vector to generate RNAi constructs, namely *MdERF1B*-RNAi and *MdMYC2*-RNAi. We inserted recombinant vectors in *A. tumefaciens* strain LBA4404 cells, and these cells were then used for infection of ‘Orin’ calli. Then, we cultivated calli on MS selective medium that contained 250 mg L^−1^ carbenicillin, 100 mg L^−1^ hygromycin-B or 50 mg L^−1^ kanamycin. Three successfully infected callus lines were selected as three biological replicates for determining gene and anthocyanin levels.

### Fruit injection assay

We cloned the 342-bp *MdERF1B* CDS and the 369-bp *MdMYC2* CDS into *Tobacco rattle virus* (TRV) vectors to generate antisense viral vectors, namely TRV-*MdERF1B* and TRV-*MdMYC2* [44]. The overexpression vectors pRI-*MdERF1B* and pRI-*MdMYC2* were constructed as described in the preceding section. We then transfected recombinant plasmids into the *A. tumefaciens* LBA4404 cells. In this work we conducted fruit injection assays according to Li *et al*.’s method [ 45]. The injected fruits were stored under constant light (20 000 lux) at 24°C for 5 days. Ten infected fruits were included as biological replicates in analyses of gene expression and anthocyanin content.

### Yeast one-hybrid assay

We cloned the *MdMYC2*, *MdERF1B*, and *MdEIL1* CDSs into separate pGADT7 vectors to generate the pGAD-*MdMYC2*, pGAD-*MdERF1B*, and pGAD-*MdEIL1* recombinant vectors, respectively. We cloned *MdMYC2* and *MdERF1B* promoter sequences into pHIS2 vector. Yeast strain Y187 cells were then transfected with the recombinant vectors. We then examined interactions in the transfected yeast cell strain using SD/−Trp/−Leu/−His media that contained 140 mM 3-AT. Yeast Y187 cells transformed with the empty pGADT7 vector served as controls.

### Electrophoretic mobility shift assay

We cloned *MdEIL1* and *MdERF1B* CDSs into the His-tag-containing pET-32a(+) vector, and inserted the recombinant plasmids in BL21 (DE3) cells of *Escherichia coli* to produce fusion proteins, followed by purification with a His-labeled Protein Purification Kit (CWbio, Beijing, China). Biotin-labeled probes were synthesized by Sangon Biotechnology Co., Ltd. (Shanghai, China). We prepared 3′ biotin-tagged double-stranded DNA (dsDNA) probes for DNA oligos in 5× annealing buffer (Beyotime, Shanghai, China). A LightShift Chemiluminescent EMSA Kit (Thermo Scientific, Waltham, MA, USA) was used for EMSAs.

### Chromatin immunoprecipitation–quantitative PCR

As previously described, we transfected apple calli using recombinant plasmids pRI-*MdERF1B* and pRI-*MdEIL1*. Then, we conducted ChIP-qPCR assays with an EZ-ChIP Kit (Millipore/Upstate, Temecula, CA, USA) and an anti-GFP antibody (Abmart, Shanghai, China) according to a previous description by Wang *et al*. [46]. Then, we used qPCR data to determine the amount of immunoprecipitated chromatin. All ChIP assays were performed in triplicate.

### Luciferase reporter assay

We cloned *MdERF1B*, *MdJAZ5*, *MdJAZ10*, and *MdEIL1* CDSs into the pGreenII 62-SK vector, and *MdMYC2* and *MdERF1B* promoters into the pGreenII 0800-LUC vector [47]. The recombinant vectors were then incorporated into *A. tumefaciens* LBA4404 cells, with P19 as a helper plasmid. Tobacco (*N. benthamiana*) leaf samples were transiently transformed with the *A. tumefaciens* LBA4404 cells. LUC activity was measured with the NightOWL II LB 983 In Vivo Imaging System (Berthold Technologies, Bad Wildbad, Germany). A pGreenII 0800-LUC vector including a renilla luciferase (REN) gene controlled by the 35S promoter was used as a positive reference.

### Yeast two-hybrid experiment

We cloned *MdJAZ* and *MdMYC2* CDSs into the pGADT7 vector. Given potent self-activation of MdERF1B, an *MdERF1B* fragment (55–591 bp) lacking an autoactivation domain [32] was inserted into the pGBKT7 vector. Then, we inserted recombinant plasmids in Y2H Gold yeast cells. Protein interactions were screened with X-α-galactose-containing SD/−Trp/−Leu/−His/−Ade medium.

### Pull-down experiment

We cloned *MdERF1B* CDS into His-tag-containing vector pET-32a(+), and cloned *MdJAZ5* and *MdJAZ10* CDSs into glutathione *S*-transferase (GST)-tag-containing vector pGEX4T-1. Then, BL21 (DE3) *E. coli* cells were transfected with recombinant plasmids to produce fusion proteins, followed by purification using the His-labeled Protein Purification Kit (Clontech, Palo Alto, CA, USA). A western blot assay with anti-GST and anti-HIS antibodies (Abmart) was performed to detect the fusion proteins.

### Bimolecular fluorescence complementation assay

We cloned the *MdERF1B* CDS into the pSPYNE vector and the *MdJAZ5* and *MdJAZ10* CDSs into the pSPYCE vector. Then, we inserted the recombinant plasmids in *A. tumefaciens* strain LBA4404 cells, followed by transfection into onion epidermal cells. Following 2 days of co-cultivation in dark at 24°C, yellow fluorescent protein (YFP) fluorescence was observed with the DS-Ri2 microscopic imaging system (Nikon Corporation, Tokyo, Japan).

### Protein degradation assay

We utilized the Plant Protein Extraction Kit (CWbio) to extract proteins from ‘Orin’ calli. The extracts were separately incubated with purified MdJAZ5-GST and MdJAZ10-GST fusion proteins at 24°C for 0, 2, 4, or 6 hours. A western blot (WB) assay was conducted on samples by employing the anti-GST antibody.

## Acknowledgements

We thank Liwen Bianji (Edanz) (www.liwenbianji.cn) for editing the English text of a draft of the manuscript. The current work was funded by the National Key Research and Development Program of China (2018YFD1000105), the National Natural Science Foundation of China (31872940), and the Agricultural Improved Seed Project of Shandong Province (2019LZGC008 and 2021LZGC024).

## Author contributions

S.-Q.F. designed the research. S.W., L.-X.L., Y.F., D.L. and Z.H.Z performed the experiments. X.-S.C. and S.-Q.F. analyzed the data. S.-Q.F., S.W. and Z.-L.M. wrote the manuscript.

## Data availability

All data supporting the findings of this study are available within the article and its supplementary data.

## Conflict of interest

The authors declare that they have no competing interests.

## Supplementary data

Supplementary data is available at *Horticulture Research* online.

## Supplementary Material

Web_Material_uhac142
