# Supplementary material for: MdERF1B–MdMYC2 module integrates ethylene and jasmonic acid to regulate the biosynthesis of anthocyanin in apple
Source: Hortic Res. 2022 Jun 23;9:uhac142. doi: 10.1093/hr/uhac142 (PMC9437725; doi:10.1093/hr/uhac142)
Supplement: Web_Material_uhac142 [file web_material_uhac142.docx]

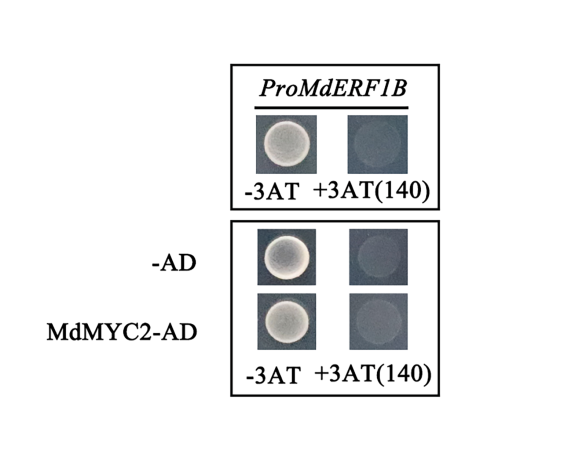


**Fig. S1: Y1H experiment indicated no binding of MdMYC2 to *MdERF1B* promoter.** The 3-AT content was 140 mM. The *MdERF1B* promoter and the empty pGAD vector were applied as NCs.


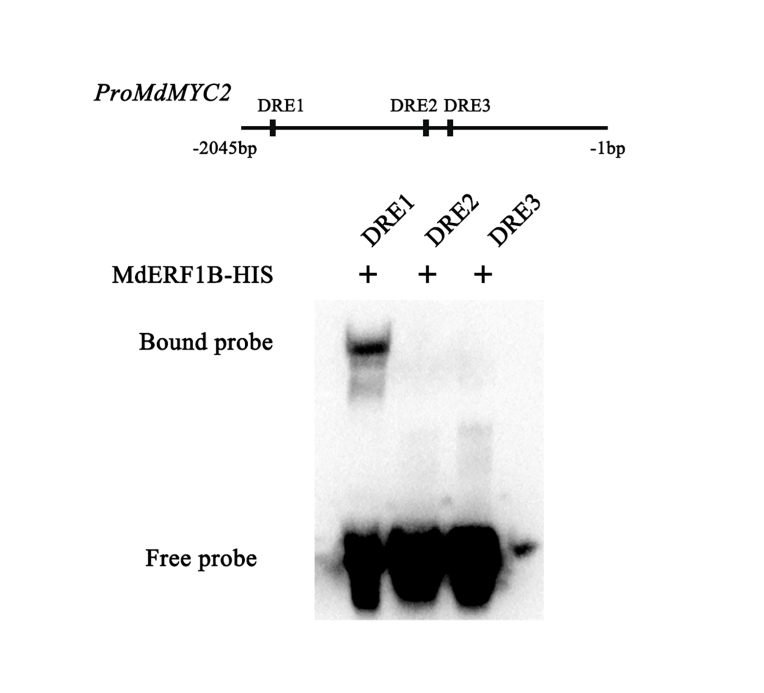


**Fig. S2: EMSAs revealed that MdERF1B only binds to DRE1 in the *MdMYC2* promoter.** Black rectangular boxes represent DRE1–3 (CCGAC) sites in the *MdMYC2* promoter.


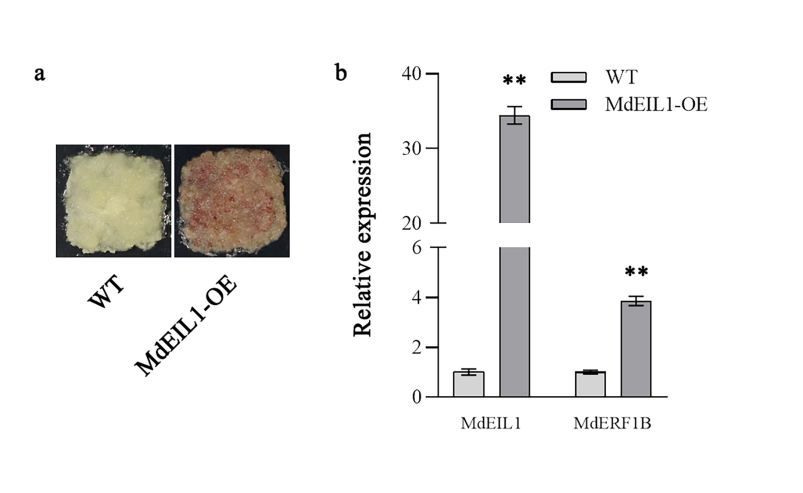


**Fig. S3: MdEIL1 activated anthocyanin synthesis and *MdERF1B* expression in apple ‘Orin’ calli.** a Phenotype of *MdEIL1*-OE calli cultured at 16 °C under light (20,000 lux) for 10 days. b *MdEIL1* and *MdERF1B* expression levels in calli. WT: wild-type control; *MdEIL1*-OE: *MdEIL1-*overexpressing calli. Error bars represent the SE from 3 replicates. ***P* < 0.01 (Student’s *t*-test).


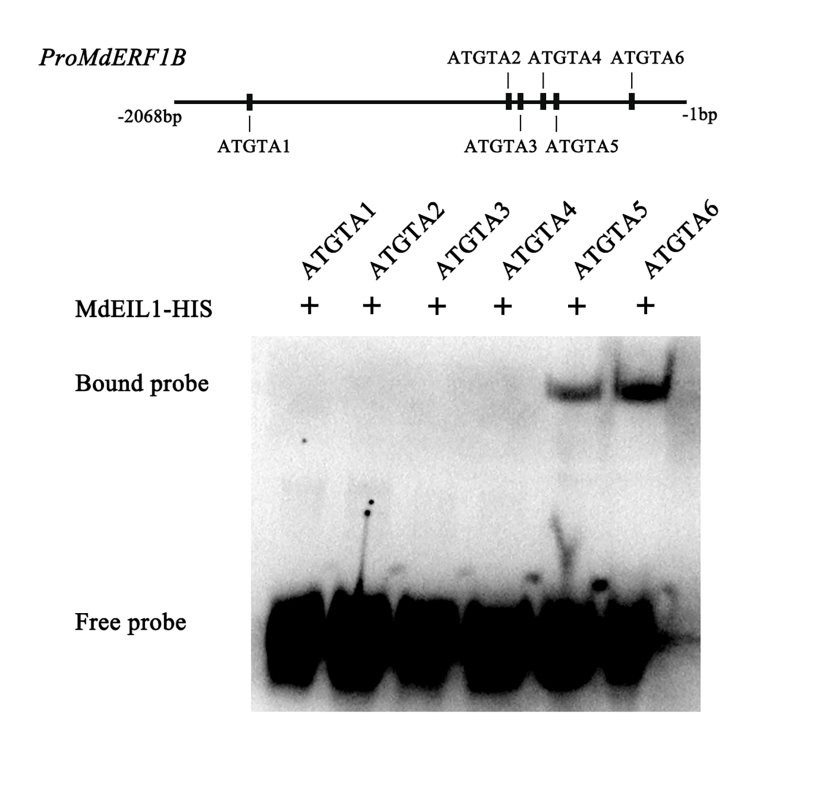


**Fig. S4: EMSAs indicated the binding of MdEIL1 to ATGTA5 and ATGTA6 in the *MdERF1B* promoter.** Black rectangular boxes represent the ATGTA1–6 sites in the *MdERF1B* promoter.


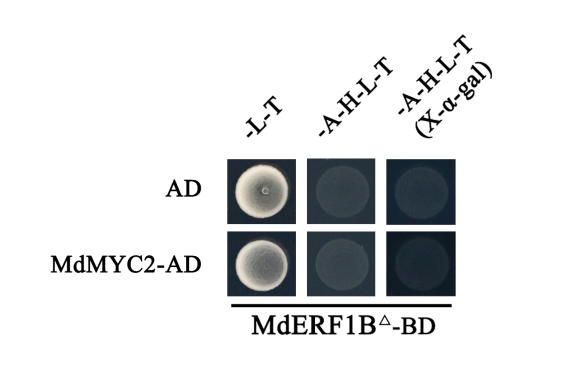


**Fig. S5: Yeast two-hybrid assay results demonstrated that MdERF1B does not interact with MdMYC2.** The empty pGAD vector served as the NC.


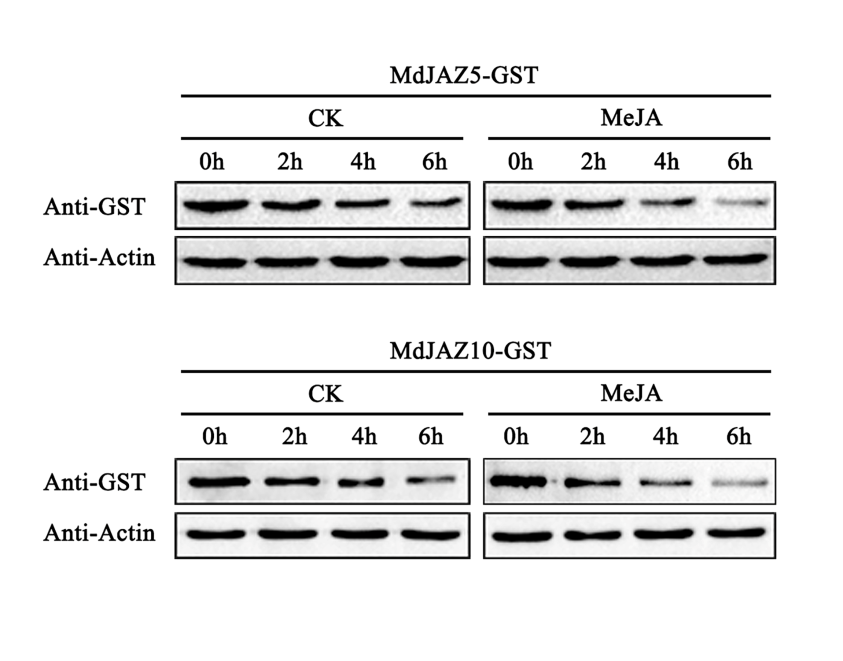


**
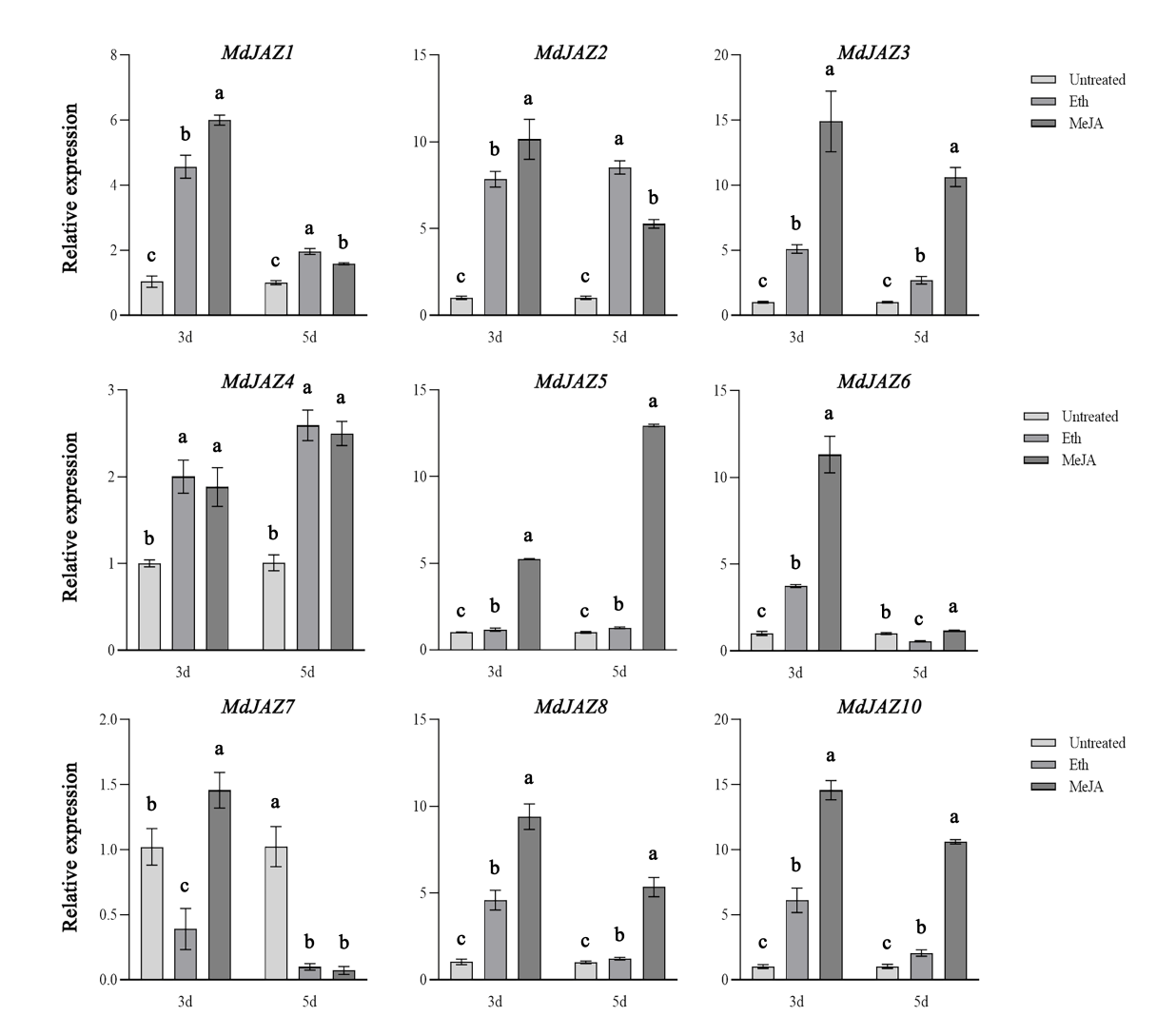
Fig. S6: Degradation of the MdJAZ5-GST (a) and MdJAZ10-GST (b) fusion proteins in response to MeJA treatment.** 100 µM MeJA was used to treat proteins extracted from WT apple calli or not, with actin being an internal control.

**Fig. S7: *MdJAZ* gene expression in apple calli treated with 100 µM MeJA and 1,000 mg∙L^−1^ ethephon at 3 and 5 days post-treatment.** Control, untreated calli; Eth, calli exposed to ethephon; MeJA, calli exposed to MeJA. Error bars represent the SE from 3 replicates. Different letters represent significant differences  (*P*< 0.05, Tukey’s test).
